# Exploiting NK Cell Surveillance Pathways for Cancer Therapy

**DOI:** 10.3390/cancers11010055

**Published:** 2019-01-08

**Authors:** Alexander David Barrow, Marco Colonna

**Affiliations:** 1 Department of Microbiology and Immunology, Peter Doherty Institute for Infection and Immunity, University of Melbourne, Melbourne, VIC 3000, Australia; 2 Department of Pathology and Immunology, Washington University School of Medicine, St. Louis, MO 63110, USA

**Keywords:** cancer, NK cell, cytotoxicity, activation, inhibition, receptor, ligand, antibody, ADCC, immunotherapy

## Abstract

Natural killer (NK) cells can evoke potent anti-tumour activity. This function is largely mediated through a battery of specialised cell-surface receptors which probe the tissue microenvironment for changes in surface and secretory phenotypes that may alert to the presence of infection or malignancy. These receptors have the potential to arouse the robust cytotoxic and cytokine-secreting functions of NK cells and so must be tightly regulated to prevent autoimmunity. However, such functions also hold great promise for clinical intervention. In this review, we highlight some of the latest breakthroughs in fundamental NK cell receptor biology that have illuminated our understanding of the molecular strategies NK cells employ to perceive malignant cells from normal healthy cells. Moreover, we highlight how these sophisticated tumour recognition strategies are being harnessed for cancer immunotherapies in the clinic.

## 1. Introduction

Natural Killer (NK) cells are large granular lymphocytes that develop from an early innate lymphoid precursor (EILP) in the bone marrow and are recognised as the founding member of the Innate Lymphoid Cell (ILC) family. Both NK cells and group 1 ILCs (ILC1) express the transcription factor T-bet and can secrete large amounts of IFN-γ and TNF-α following cellular activation. However, in comparison to ILC1, NK cells are renowned for their potent cytotoxic properties and have the ability to spontaneously lyse tumour cells by ‘natural’ cellular cytotoxicity or via antibody-dependent cellular cytotoxicity (ADCC). IFN-γ also possesses tumour cytostatic and cytotoxic properties and can arrest tumour cell proliferation, tumour angiogenesis, and multistage carcinogenesis [1], as well as induce the cell-surface expression of ligands for NK cell receptors on cancer cells further enhancing tumour immunosurveillance [2,3]. Moreover, IFN-γ facilitates classical macrophage activation in addition to influencing subsequent adaptive immune responses [4,5]. Thus, NK cell activity is associated with resistance to various intracellular pathogens as well as a more favorable prognosis and lower incidence of cancer [6,7,8,9,10]. The ability to promote the anti-tumour functions of NK cells could therefore provide powerful therapeutic tools for cancer immunotherapy.

**Figure 1:**
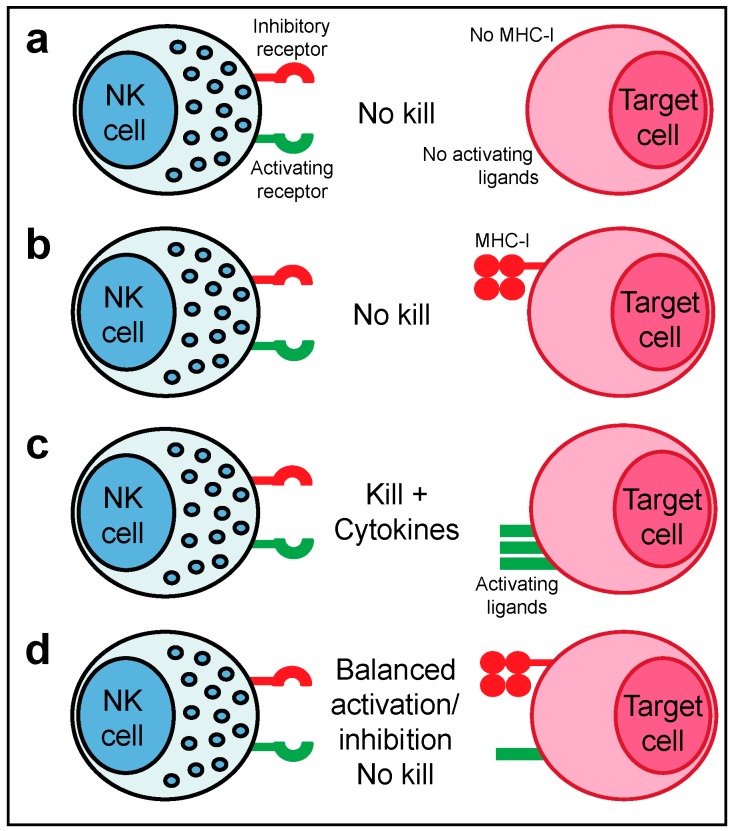
Molecular basis for ‘missing-self’ and ‘induced-self’ recognition by NK cells: (**a**) NK cells do not respond if either the ligands for activating receptors or ligands for inhibitory receptors e.g., MHC-I are not expressed on target cells; (**b**) If MHC-I ligands engage inhibitory receptors, such as KIR or NKG2A, on target cells in the absence of ligands for activating receptors then no cytotoxicity is observed; (**c**) Downregulation of MHC-I and expression of ligands for activating receptors results in robust NK cell cytotoxicity and secretion of cytokines, such as IFN-γ and TNF-α; (**d**) NK cell responses are regulated by a balance of activating and inhibitory signalling, such that sufficient expression of MHC-I can prevent target cell cytotoxicity even if there is low level expression of activating receptor ligands. In humans, classical MHC-I comprises Human Leukocyte Antigen (HLA)-A, -B and -C molecules and non-classical MHC-I comprises HLA-E, -F, and -G.

NK cell function is tightly regulated by a family of activating and inhibitory receptors that bind to cell-surface and extracellular secreted ligands (Figure 1). For example, according to the now classical model of NK cell activity, the ligands for inhibitory receptors are constitutively expressed by healthy cells e.g., Major Histocompatibility Complex class I molecules (MHC-I) but are lost upon infection or cellular transformation. Conversely, activating receptors, such as NKG2D, can engage host-encoded ligands that are induced upon infection or cellular transformation (termed ‘induced self recognition’) [11]. The loss of inhibitory ‘checkpoints’ allows activating signals to predominate and forms the basis for ‘missing-self recognition’ (Figure 1). Therapeutically manipulating the balance of signalling from activating and inhibitory receptors on NK cells as well as other immune cells holds great promise for cancer immunotherapy, as exemplified by the success of checkpoint blockade.

Despite possessing many clinically desirable anti-tumour properties, NK cell-based immunotherapies have yet to achieve full potential in the clinic. Several barriers to the successful development of NK cell-based cancer therapies exist particularly for solid tumours that establish an immunosuppressive tumour microenvironment [12]. However, a recent meta-analysis, which analysed gene expression in ~18,000 human tumours across 39 malignancies, showed that the expression of genes for the NK cell family receptors, such as members of the Killer lectin-like receptor family e.g., *KLRG1* (see also: https://precog.stanford.edu/index.php), are associated with a more favourable prognosis [13]. In this review, we will highlight the different cell-surface receptors NK cells employ to respond to malignant cells and how these various innate recognition systems can be exploited for cancer immunotherapy.

## 2. Killer Cell Ig-Like Receptors (KIR)

The development of the ‘missing-self’ hypothesis was based on the observation that NK cells spontaneously lyse syngeneic target cells lacking expression of MHC-I [14]. This mode of MHC-I-dependent recognition explains why NK cells can attack virus-infected or cancer cells that have downregulated MHC-I to evade recognition by CD8^+^ T cells, whereas healthy autologous cells expressing MHC-I are spared from attack. In humans, the main inhibitory receptors for ‘self’ MHC-I are the inhibitory KIR and CD94-NKG2A [15] (in mice Ly49 receptors are the functional equivalent of KIR [16]). However, the missing-self hypothesis failed to explain why some autologous cells that lack MHC-I expression are protected from NK cytotoxicity e.g., human erythrocytes. The identification and characterisation of several activating NK cell receptors that sense ligands induced upon cellular stress or infection led to the proposal of the ‘induced-self’ recognition model, which states that NK cell triggering also requires the expression of ligands for activating NK cell receptors. Consequently, it is now well accepted that the activation of mature NK cells is dependent on a balance of activating versus inhibitory signals with full NK effector activity only triggered once a threshold of inhibitory signalling is overcome (Figure 1).

### 2.1. NK Cell Education

More recently, evidence has accumulated that the functional capabilities of NK cells are tuned to the levels of MHC-I expression, both in cis and in trans, as part of a process of NK cell maturation termed ‘education’: NK cells expressing inhibitory receptors for MHC-I respond efficiently to activation stimuli in comparison to NK cells lacking MHC-I receptors that respond poorly. The mechanism of NK cell education is not very well understood but permits appropriate NK cell responses to host cells lacking MHC-I and ensures NK cell effector functions are adapted to the host in which they develop. For example, when NK cells develop in mice or patients deficient in MHC-I, the hosts do not develop autoimmunity and the NK cells are hyporesponsive to in vitro stimulation [17,18,19]. To add to this complexity, the genes encoding KIRs and MHC-I molecules are polymorphic and polygenic and encoded on different haplotypes that segregate independently leading to diverse KIR/HLA genotypes [20]. Due to the variegated expression of KIR, a fraction of NK cell clones may express KIR that lack cognate MHC-I ligands and therefore cannot undergo NK cell education and are rendered hyporeactive [21]. The inherited KIR/HLA genotype may therefore profoundly influence the education and functional capacity of NK cells [22]. However, as a consequence of this system, NK cells not only have the ability to carefully distinguish between normal and aberrant cells but also allogeneic cells due to their exquisite ability to sense HLA polymorphisms [23].

### 2.2. KIR and Haematopoietic Stem Cell Transplantation (HCST)

The ability of NK cells to perceive allogeneic cells is thought to play a critical role for patients with acute myelogenous leukaemia (AML) receiving HLA-haploidentical haematopoietic stem cell transplantation (HCST) from an NK-alloreactive donor. In this transplantation setting, the recipient shares only an HLA haplotype with the donor (usually a parent in the case of a paediatric patient) and is utilised for high risk AML patients in the absence of an HLA-compatible donor. Thus, haploidentical HCST requires e.g., the extensive depletion of αβ T cells ex vivo to avoid severe graft versus host disease. However, in the HLA-haploidentical HCST setting, the absence of HLA ligands for donor inhibitory KIR has been associated with a lower relapse and improved survival in AML patients. Such patients can develop a significant ‘graft versus leukaemia’ (GVL) response in which the donor-derived NK cells remain unrestrained by inhibitory HLA ligands expressed on the recipient’s AML cells [24,25,26].

This GVL effect was thought to be attributed to the killing of ‘missing self’ targets by fully educated NK cells. However, NK cell alloreactivity has been reported to occur even in HLA-matched HCST [27]. These data indicate that uneducated NK cells expressing KIR for HLA ligands that are not present in either the donor or the recipient (i.e., ‘non-self’ MHC-I) may achieve functional competence in HCST [28], perhaps due to the pro-inflammatory microenvironment following transplantation [29]. The NK cell repertoire is also known to be shaped by CMV infection, which frequently occurs in patients that have undergone HSCT [30], and can give rise to a population of CD56^dim^CD57^+^NKG2C^+^ adaptive NK cells that produce more IFN-γ and TNF-α following target cell recognition [31]. Thus, it may be possible that NK cells could undergo expansion in response to virus reactivation to contribute to a GVL effect [32].

Allogeneic NK cell therapy has also been shown to be beneficial in targeted antibody (Ab) therapies, such as anti-GD2 therapy for the treatment of neuroblastoma and anti-CD20 therapy for the treatment of lymphoma [33,34,35]. Both educated and uneducated NK cells actively kill neuroblastoma target cells with anti-GD2 Ab via ADCC, but educated NK cells were selectively inhibited by MHC-I present on target cells [33]. These studies show that during the course of cancer, uneducated NK cells may attain functional activity that is clinically beneficial and challenges the perception of a lack of education and hyporeactivity. Moreover, for fully ‘educated’ NK, the presence of self MHC-I on cancer cells may not necessarily predict loss of NK cell effector function due to differences in inhibitory KIR binding due to HLA allelic diversity. For example, compared to donor NK cells with strong KIR3DL1 binding HLA allotypes, donor NK cells expressing KIR3DL1 with weak or no binding to HLA-B allotypes were associated with improved control for AML patients and for neuroblastoma patients receiving anti-GD2 Ab therapy [36,37]. Taken together, these studies suggest that the tuning of NK cell functional activity to MHC-I levels during the NK cell education process may be sufficient to prevent NK cell autoreactivity during steady state but can be overridden in stressful conditions e.g., malignancy, microbial infection, or upon treatment with therapeutic Abs, such as anti-GD2 therapy.

## 3. Monoclonal Antibodies for Cancer Immunotherapy

Recent studies indicate that monoclonal antibodies (mAbs) can be designed to elicit or enhance existing anti-tumour immune responses. Such ‘checkpoint blockade mAbs’ rely on the principle of disrupting suppressive signalling from inhibitory receptors that are expressed by killer lymphocytes [38,39,40]. Inhibitory receptors normally function to limit tissue immunopathology during acute viral infections [41,42,43] but may also facilitate T cell exhaustion during chronic viral infections and anti-tumour immune responses [44,45]. Checkpoint inhibitory receptors include the cytotoxic T lymphocyte-associated protein 4 (CTLA4) [46,47,48] and programmed cell death 1 (PD-1) [49,50,51] or their cognate ligands, such as PD-1 ligand (PD-L1) [52,53]. However, resistance to these first generation immune checkpoint inhibitors frequently leads to treatment failure, thus providing the necessary impetus to discover new candidates for checkpoint blockade [54].

### 3.1. PD-1

Monoclonal antibodies to checkpoint inhibitory receptors have revolutionised cancer treatment and a variety of combinatorial approaches are now being tested in clinical trials. The therapeutic efficacy of PD-1 and CTLA-4 checkpoint blockade is thought to be mediated largely through the rescue of exhausted tumour-specific T cells and subsequent restoration of their effector functions. Few studies have reported PD-1 expression by NK cells. However, a link between NK cell expression of PD-1 and CMV serostatus exists [55] and PD-1 expression on NK cells from multiple myeloma patients has also been described [49].

Many cancer types exhibit low expression of MHC-I and/or low neoantigen burden that should render tumour cells refractory to CD8^+^ T cell recognition. High levels of PD-L1 expression have also been observed for tumours with low MHC-I expression [50,56,57,58]. Intriguingly, some of these latter types of cancers are responsive to PD-1/PD-L1 blockade even when the tumours were defective in MHC-I expression suggesting immune cells other than cytotoxic T cells can play a role [59].

Recently, PD1 was found to be expressed on NK cells in transplantable, spontaneous and genetically induced tumour models [60]. Moreover, PD-L1 expression on cancer cells resulted in reduced NK cell responses and precipitated more aggressive tumours in vivo. PD1 and PD-L1 blockade was subsequently found to induce a strong NK cell response demonstrating that NK cells as well as T cells mediate the effects of PD1/PD-L1 blockade immunotherapy, which may be critical in scenarios where tumours express low levels of MHC-I and high levels of PD-L1 [60]. 

### 3.2. NKG2A

NKG2A is a lectin-like inhibitory receptor that is expressed as a heterodimer with CD94 on NK cells and activated CD8^+^ T cells. The CD94-NKG2A heterodimer binds to the non-classical MHC-I molecule HLA-E [61] and Qa-1 in mice [62]. Both HLA-E and Qa-1 bind to peptides derived from the signal sequence of classical MHC-I molecules (as well as peptides derived from the CMV UL40 gene in the case of HLA-E) and engage with NKG2A to inhibit NK and T cell effector functions [62,63,64,65,66]. Blocking the NKG2A/HLA-E interaction therefore has the potential to restore NK cell and CD8^+^ T cell cytotoxicity of tumour cell targets.

Recently, high dimensional mapping of tumour-infiltrating lymphocytes (TILs) using 36 colour Cytof revealed that cancer vaccines can induce the expression of NKG2A on a population of CD103^+^ effector CD8^+^ T cells. IFN-γ also upregulated Qa-1 and HLA-E on murine and human tumour cells, respectively, and blocking NKG2A converted cancer vaccines into effective therapies in four different solid tumour models (TC-1 lung epithelial tumour, B16F10 melanoma, RMA T cell lymphoma, and MC38 colon carcinoma) [67]. Interestingly, the expression of Qa-1 by tumour cells, and not stromal or immune cells, was required for this additive effect [67]. Moreover, the humanised anti-NKG2A mAb, monalizumab, unleashed the activity of both CD8^+^ T and NK cells in two murine lymphoma tumour models (A20 B cell lymphoma and RMA-Rae1β) in combination with anti-PD-1/PD-L1 Ab blockade [68]. In addition, a combination of monalizumab and cetuximab, an anti-EGFR Ab, led to a 31% objective response rate (i.e., a proportion of patients a reduction in tumour size for a predefined amount and for a minimum time period) in a clinical trial for head and neck squamous cell carcinoma patients [68].

### 3.3. T-Cell Immunoglobulin and Mucin-Domain-Containing-3 (Tim-3)

Tim-3 is expressed by activated and exhausted T cells and NK cells and has been characterised as a negative regulator of T cell-mediated immune responses. Tim-3 has been reported to bind to several ligands; galectin-9, phosphatidylserine on apoptotic cells, high mobility group box 1 (HMGB1), and CEACAM-1 [69,70,71,72]. Galectin-9 was reported to inhibit the effector functions of T helper 1 (Th1) cells by inducing Tim-3-dependent calcium signalling, aggregation, and cell death [70]. 

Tim-3 does not carry any Immunoreceptor Tyrosine-based Inhibition Motifs (ITIM) or Immunoreceptor Tyrosine-based Switch Motifs (ITSM) in its cytoplasmic tail. Instead, Tim-3 has five conserved tyrosine residues in its cytoplasmic tail with Y256 and Y263 reported to recruit HLA-B-associated transcript 3 (Bat3) [73]. Bat3 binds to Tim-3 in steady state and recruits catalytically active Lck, which promotes T cell signalling and prevented Tim-3-mediated cell death [73]. Galectin-9 and CEACAM-1 binding to Tim-3 induced the Y256 and Y263 phosphorylation, resulting in disassociation of Bat3 and SH2 domain-dependent recruitment of Fyn, which was suggested to promote Tim-3 inhibitory signalling [73]. However, other groups could find no evidence of an interaction between human or mouse Tim-3 and galactin-9 [74] and the crystal structure of a heterodimer between the V domains of CEACAM-1 and Tim-3 has since been withdrawn [69]. Other groups have reported Tim-3 interactions with Fyn and the p85 sub-unit of phosphatidylinositol 3-kinase [75] as well as downstream Akt/mTOR signalling for optimal T cell effector responses in vivo [76].

On NK cells, Tim-3 has also been reported to have either activating or inhibitory functions depending on the context. For example, blockade of galactin-9 reduced NK cell secretion of IFN-γ when co-cultured with AML target cells, suggesting Tim-3 is an activating receptor [77]. In contrast, cross-linking with anti-Tim-3 antibodies resulted in NK cell inhibition [78]. Blockade of Tim-3 can rescue exhausted NK cells from patients with advanced melanoma and lung adenocarcinoma and resulted in enhanced NK cell cytotoxicity and IFN-γ production [79,80,81].

Tim-3 is constitutively expressed on several myeloid lineages, such as macrophages and dendritic cells (DC). Therapeutic Abs to Tim-3 may therefore have a strong impact on the antigen presenting functions of these cells, particularly since Abs to Tim-3 have been shown to induce DC activation [82]. Given that the role of Tim-3 in regulating the effector functions in T and NK cells remains to be fully clarified and the potential for anti-Tim-3 Abs to activate myeloid cell function, it will be interesting to understand the mechanism of action for therapeutic approaches that target Tim-3. The therapeutic Tim-3 blocking mAb TSR-022 is currently in phase 1 clinical trials for patients with advanced solid tumours [83].

### 3.4. T-Cell Immunoreceptor with Immunoglobulin and Immunoreceptor Tyrosine-Based Inhibition Motif Domains (TIGIT)

TIGIT is an inhibitory receptor that binds to CD155, also known as the poliovirus receptor (PVR), and to CD112, also known as Nectin-2 and poliovirus receptor-like 2 (PVRL2) [84]. PVR and Nectin-2 are also ligands for the activating NK cell receptor CD226, also known as DNAM-1 [85]. Thus, TIGIT and DNAM-1 can compete for binding to PVR and Nectin-2 which are highly expressed on tumour cells and are also upregulated by exposure to cytokines, such as IFN-γ and TNF-α [3].

TIGIT contains an ITIM and immunoreceptor tyrosine tail (ITT)-like motifs in its cytoplamsmic tail and ligand-engagement of TIGIT can result in the recruitment of the SH2 domain-containing inositol 5′-phosphatase (SHIP) leading to downregulation of the PI3 kinase, MAPK and NF-κB signalling pathways and inhibition of NK cell cytotoxicity and cytokine secretion [84,86]. TIGIT therefore counterbalances NK cell activation mediated by DNAM-1, which is reversed by Ab blockade of TIGIT [84]. Interestingly, TIGIT blockade can also render adaptive NK cells resistant to inhibition by myeloid suppressor cells [87]. Antibody blockade of TIGIT and the PD-1/PD-L1 axis enhanced tumour cell clearance by CD8^+^ T cells [88,89] and significantly prolonged control of myeloma in a mouse model of autologous stem cell transplantation [90]. Despite efficacy in pre-clinical tumour models, whether individual blockade of TIGIT or in combination with other checkpoint therapies can enhance NK cell effector function for the generation of effective anti-tumour response in human cancer patients remains to be demonstrated. 

### 3.5. Interleukin-1 Receptor 8 (IL-1R8)

Interleukin-1 receptor 8 (IL-1R8, also known as single immunoglobulin (Ig) IL-1R-related receptor, SIGIRR) is a member of the IL-1 receptor (IL-1R) family. IL-1R8 acts as a negative regulator of IL-1R family and Toll-like receptor function [91]. IL-1R8 is a 410aa protein with a single Ig-like domain compared to other IL-1R family members that encode three Ig-like domains, a transmembrane domain, and a cytoplasmic Toll-IL-1 resistance (TIR) domain followed by an uncharacteristically long stretch of 95 amino-acid residues. The absence of two highly conserved S447 and Tyr536 residues (replaced by Cys222 and Leu305) in the IL-1R8 TIR domain suggests an unconventional mechanism of intracellular signalling. IL-1R8 can be recruited to signalling complexes where it competes for the formation of Myd88 dimers via its TIR domain, thus blocking the recruitment of cytoplasmic signalling components and inhibiting downstream activation of NF-κB and JNK [92]. In addition, the ectodomain of IL-1R8 was also shown to block the dimerisation of IL-1R1 and IL-1R3 as well as inhibit ST2 signalling [92,93]. Moreover, IL-1R8 pairs with IL-18Rα to form a receptor for the anti-inflammatory cytokine, IL-37 [94]. IL-1R8 deficiency is associated with intestinal inflammation and increased susceptibility to colitis-associated cancer development [95]. IL-1R8 deficiency also induced an earlier and more severe expansion of B cell clones and reduced survival in the Eμ-TCL1 transgenic mouse model of chronic lymphocytic leukaemia [96]. Thus, IL-1R8 may play a protective role in some malignancies that thrive upon inflammation.

Murine and human NK cells express high levels of IL-1R8 which is acquired during NK cell differentiation and deficiency in IL-1R8 results in higher numbers of mature NK cells in blood and tissues, such as bone marrow, spleen, and liver [97]. IL-1R8^−/−^ NK cells have a more activated phenotype with higher expression levels of activating receptors, IFN-γ, and cytotoxic mediators, such as granzyme B and Fas ligand, and more readily degranulated compared to wild-type NK cells. Mechanistically, IL-1R8 suppressed IL-18 signalling which is a key cytokine for NK cell activation [98,99]. In IL-1R8^−/−^ mice, tumour burden was significantly reduced in models of hepatocellular carcinoma and lung and colon metastasis. Moreover, the adoptive transfer of Il1r8^−/−^ NK cells provided sufficient protection in the metastasis models suggesting that blockade of IL-1R8 may represent a therapeutic approach to enhance NK cell activity and promote anti-tumour activity in the clinic [97]. However, caution may be warranted for malignancies in which IL-1R8 may play a protective role [95,96].

### 3.6. Sialic Acid Binding Immunoglobulin-Like Lectins (Siglecs)

Sialic acids are sugars that are incorporated into the periphery of cell-surface glycans [100]. The Sialic acid-binding Ig-like lectins (Siglecs) are a multi-gene family of cell-surface activating and inhibitory receptors expressed by lymphoid and myeloid cells in mammals, amphibians, and fish [101,102]. Consequently, the sialic acid content of host cell-surface glycans has the potential to regulate immune responses. Tumour cells characteristically express a high density of sialic acid enriched cell-surface glycoproteins arising from epigenetic or genetic disruption of glycan synthesis pathways [103]. The resulting ‘hypersialylated’ tumour cell-surface phenotype is associated with poor patient survival and decreased immunogenicity in a range of tumours [103]. 

NK cells constitutively express Siglec-7 and a subset of CD56^dim^ NK cells was shown to express Siglec-9 [104,105,106]. Evidence has accumulated that NK cells may play a direct role in selecting for the hypersialylated cancer cell-surface phenotype. For example, tumours that develop in *Ifng^−/−^* mice fail to develop a hypersialylated cell-surface phenotype and a correlation exists between tumour cell-surface sialylation and resistance to NK cell-mediated cytotoxicity [107,108,109]. Cell-surface hypersialylation may therefore provide a selective advantage for tumour cells under evolutionary selective pressure from killer lymphocytes by directly engaging inhibitory Siglecs. In support of this, one study found sialic acid ligands for Siglec-7 and -9 were expressed by a wide range of primary tumours and inhibited NK cell activation [105]. Interestingly, a subset of circulating Siglec-9^+^ CD56^dim^ NK cells with enhanced chemotactic responses was reduced in patients with colon adenocarcinoma and malignant melanoma [105].

Therapeutic interventions that target tumour-associated sialosides from engaging inhibitory Siglec receptors expressed by killer lymphocytes may provide a promising new avenue for cancer immunotherapy. Recently, polymorphisms in the gene encoding Siglec-9 were associated with the development of lung and colorectal cancer [110]. Siglec-9 was also upregulated on a population of tumour-infiltrating cytotoxic T cells from non-small cell lung cancer (NSCLC), colorectal, and ovarian cancer patients and T cell expression of Siglec-9 was associated with reduced survival in NSCLC patients. In mouse tumour models, transgenic expression of Siglec-9 enhanced tumour growth. Siglec-E is the functional paralogue of Siglec-9 in mice. Targeting of the tumour sialoglycan by exchanging the inhibitory signalling domain of Siglec-E with that of the activating Siglec-16 receptor resulted in enhanced anti-tumour immunity [110,111].

## 4. Augmenting Activating NK Cell Receptor Pathways

Another intuitive approach to cancer immunotherapy is to augment NK cell activation pathways. Most therapeutic mAbs promote anti-tumour responses either by directly triggering ADCC or by targeting co-stimulatory receptors expressed on the surface of NK cells. Other approaches target the ligands for activating NK cell receptors, either by preventing their shedding from cancer cells or by hindering the ability of the shed ligands to induce NK cell desensitisation. Finally, recombinant approaches are now being adopted that endow T cells and NK cells with the ability to target tumour cells directly and with enhanced signalling potential.

### 4.1. CD16

One strategy to enhance NK cell function is to exploit the ability of NK cells to recognise Ab-coated targets through CD16 to mediate the potent killing of tumour cells via ADCC [112]. CD16, also known as Fcγ receptor IIIa, FcγRIIIa, binds the Fc region of immunoglobulin G (IgG) and signals via association with the Immunoreceptor Tyrosine-based Activation Motif (ITAM)-bearing adaptors, CD3ζ and Fc receptor common γ (FcRγ) chain in NK cells [113,114]. CD16 genotypes vary in their respective affinity for the Fc region of IgG, which can dramatically influence clinical outcome. For example, NK cells expressing the CD16 158VV or 158VF genotype have lower affinity for the Fc region of rituximab (anti-CD20 mAb) than the CD16 158FF genotype [115]. CD16 is the most potent activating receptor expressed by NK cells and can readily induce potent cytotoxicity and cytokine secretion from freshly isolated NK cells [116].

CD16 activity on resting NK cells is therefore dependent on Abs produced by B cells. However, several therapeutic mAbs have now been designed that mediate their clinical effects through the induction of ADCC by resting NK cells. Moreover, CD16 can even promote ADCC from uneducated NK cells that are normally hyporesponsive [33]. The lack of inhibitory MHC-I receptors expressed by uneducated NK cells may well be a distinct advantage since MHC-I expression by cancer cells selectively inhibited ADCC by educated NK cells indicating that uneducated NK cells may play a central role in cancer patients undergoing mAb-based immunotherapies [33].

Strategies to enhance ADCC for Ab-based cancer therapies are also being formulated. NK cell activation can result in decreased CD16 cell-surface expression, which could drastically influence the efficacy of mAb-based cancer therapies [117]. The decrease in cell-surface expression was attributed to cleavage of CD16 by a disintegrin and metalloproteinase-17 (ADAM17) resulting in shedding of the CD16 receptor from the surface of NK cells. The selective inhibition of CD16 cleavage by an ADAM17 inhibitor led to increased IFN-γ production [118]. Clinical studies are now being conducted using ADAM17 inhibitors in combination with anti-CD20 rituximab after HCST in patients with diffuse large B cell lymphoma [119].

### 4.2. Signalling Lymphocytic Activation Molecules Family 7 (SLAMF7)

The SLAM family contains six members named SLAM, 2B4, Ly-9, natural killer (NK)-, T- and B-cell antigen (NTB-A), CD84 and SLAMF7 (also known as CRACC and CS1) [120]. NK cells express at least three SLAM family receptors, 2B4, NTB-A, and SLAMF7. 2B4 binds CD48 whilst SLAMF7 and NTB-A mediate homophilic adhesion. The cytoplasmic domains of SLAM receptors contain the amino acid motifs, TxYxxV/I, termed the ITSM. Engagement of SLAM family receptors results in tyrosine phosphorylation receptor of ITSMs and the recruitment of SLAM-associated protein (SAP) family of adaptors, such as SAP (also called SH2D1A or DSHP) or the EWSFli1-activated transcript-2 (EAT-2). All SLAM family members can bind SAP or EAT-2. However, SLAMF7 is unique in recruiting EAT-2 that activates the PI3-kinase and phospholipase C-γ signalling pathways in human NK cells [121].

Interestingly, SLAMF7 expression was observed in normal and neoplastic plasma cells in nearly all patients with monoclonal gammopathies of undetermined significance (MGUS), smouldering myeloma and multiple myeloma, but not in normal tissues or a variety of solid tumours [122,123]. A humanised Ab to SLAMF7, HuLuc63, exhibited NK-mediated ADCC of primary myeloma cells in vitro and anti-tumour activity in vivo that was depended on NK cells and Fc-CD16 interactions. HuLuc63 is now marketed as Elotuzumab and is one of the first mAbs to be approved for the treatment of multiple myeloma [124]. Interestingly, in addition to binding SLAMF7 on myeloma cells and engaging Fc-CD16 interactions, Elotuzumab may further enhance NK cell cytotoxicity by directly stimulating cell-surface SLAMF7 on NK cells by redirected cytotoxicity (a mechanism whereby the antibodies are immobilised e.g., by Fc receptors on target cells leaving the Fab regions free to engage activating SLAMF7 expressed by the NK cells) and may highlight the effectiveness of strategies to develop therapeutic antibodies that can target activating receptors expressed by both the cancer cells and NK cells to complement CD16 signalling and enhance ADCC [125]. 

### 4.3. Natural Killer Group 2D (NKG2D)

NKG2D is a highly conserved receptor that can either activate or co-stimulate NK cells and subsets of T cells. In humans, NKG2D transmits signals through its association with the DAP10 adaptor molecule [126,127]. The ligands for the NKG2D receptor comprise an array of proteins that are structurally related to MHC-I. In humans, the complement of NKG2D ligands (NKG2DLs) comprise the MHC-I-polypeptide-related sequence family, MICA and MICB (collectively known as ‘MIC’), and six members of the UL16-binding protein (ULBP) family that are also known as the retinoic acid early transcript (RAET) proteins (RAET1E, RAET1G, RAET1H, RAET1I, RAET1L and RAET1N), which can be expressed from various alternatively spliced transcripts [127,128,129,130,131].

In general, the expression of NKG2DLs is strictly regulated at the level of transcription, translation and post-translation in healthy tissues [132,133,134]. The human NKG2D ligand MICA was first described as a stress response molecule induced by heat shock [127] but it is now appreciated that NKG2DLs are readily induced upon infection with a wide range of different viruses [132]. NKG2DLs are also expressed on many solid tumours and leukaemias [131,135,136] and are also induced by cancer-associated pathways, such as the DNA damage response (DDR) and the expression of oncogenes [133]. Moreover, there is evidence that NKG2D mediates anti-cancer responses to solid tumours and leukaemias in vivo [137,138].

The central importance of NKG2D in mediating anti-viral and anti-tumour responses is emphasised by the various strategies that viruses and tumour cells have formulated to evade NKG2D-mediated surveillance. For example, human CMV encodes several molecules and microRNAs that prevent the expression of NKG2DLs at the infected cell-surface [132,139] and tumours can express proteases that cleave NKG2DLs from the cell-surface, or release cytokines, such as TGF-β, that downregulate NKG2D, or simply switch off the expression of NKG2DLs as they grow and metastasise [140,141,142,143]. These data strongly suggest that NKG2D participates in immunosurveillance of various forms of cellular stress and that the NKG2DLs appear to have evolved as an innate mechanism whereby a host cell might signal distress and thus mark itself for elimination by NK cells.

In terms of cancer therapy, it is well appreciated that MICA and MICB are abundantly expressed in human tumours [135]. However, high levels of circulating soluble NKG2DLs shed from the cancer cell-surface have been shown to be immunosuppressive. Soluble MIC ligands are associated with poor prognosis for multiple tumour types and a diminished response to checkpoint blockade in clinical and pre-clinical studies, most likely by inducing the endocytosis and degradation of NKG2D [135,143]. Various approaches to reinvigorate the immune response have been devised that target the generation of soluble MIC, such as targeting sequences in the α3 domain of MIC [144] or the disulphide-isomerase ERp5 that regulates the proteolytic shedding of MIC [145], as well as the removal of soluble MIC using anti-MIC monoclonal antibodies (mAbs) [146] or via plasma absorption apheresis prior to adoptive NK cell therapy [147]. The mAb-mediated clearance of soluble MIC has shown promising synergy with the IL-15 agonist ALT-803 mAb and enhanced anti-tumour responses with anti-CTLA4 checkpoint blockade therapy in clinically relevant models [148]. More recently, Ab-based inhibition of MICA and MICB shedding promoted anti-tumour immunity through the activation of NK cells through dual stimulation of the NKG2D and CD16 Fc receptor pathways [149].

In some tumour models, forced expression of the membrane-bound NKG2DLs, MICA and murine Rae-1ε, were reported to impair NKG2D function through chronic receptor stimulation [133,150,151]. Remarkably, the shed form of the high affinity murine NKG2D ligand, MULT1, induced NK cell activation and tumour rejection via a mechanism that was reported to reverse global NK cell desensitisation imposed by membrane-bound NKG2DLs expressed by tumour-associated cells [152]. 

Recent studies have also shown that soluble ligands for activating NK cell receptors, such as platelet-derived growth factor (PDGF)-DD that engages NKp44, can also stimulate NK cell activation [3]. It is likely that PDGF-DD and soluble MULT1 may induce NK cell activation via different signalling and/or cell biological mechanisms. However, these studies indicate that a model whereby soluble ligands for activating NK cell receptors are predominantly inhibitory may be over-simplified and natural variation in NK tumour surveillance systems exists. A greater understanding of how soluble ligands interact with their cognate receptors to modulate NK cell activation and generate functional anti-tumour responses is required for the rational design of novel NK cell-based cancer immunotherapies.

## 5. Recombinant Approaches to Cancer Immunotherapy

### 5.1. NKG2D Chimeric Antigen Receptors (CARs)

The use of T cells engineered to express receptors for cancer-specific antigens, such as the anti-CD19 chimeric antigen receptor (CAR), has shown encouraging promise in the treatment of heamatological malignancies resulting in remission rates of up to 90% in individuals with paediatric lymphoblastic leukaemia [153]. Conventional approaches to CAR-based cancer immunotherapy take advantage of single-chain variable fragment (scFv)-based CARs to target tumour surface antigens. However, emerging strategies to target tumour cells also include the use of NK cell receptors, such as NKG2D to target NKG2DL^+^ tumours.

Various NKG2D-based CARs have been designed either with DAP10 or with the 4-1BB or CD28 signalling modules but all in combination with CD3ζ [154]. NKG2D-CARs can bestow T cells with cytotoxic and cytokine secreting functions against tumour cell targets and control the growth of a number of tumour types in mouse models of multiple myeloma [155], ovarian carcinoma [156], osteosarcoma [157], breast cancer [158], and glioblastoma [159], and have also been adopted to enhance the activity of NK cells in osteosarcoma [160]. NKG2D-CARs are currently undergoing clinical evaluations for haematological [136] and metastatic tumours [161].

### 5.2. Bi- and Tri-Specific Killer Engagers (BiKEs and TriKEs)

Whilst recent focus has concentrated on the generation of CAR-expressing T and NK cells, such approaches are expensive and time consuming, have proven to lack efficacy for solid tumours, and are often associated with significant toxicity issues. BiKEs and TriKEs are small molecules (50–75 kDa compared to 300–450 kDa of bi- and tri-specific antibodies [162]) encoded by a single-chain variable fragment (scFv) comprised of a variable heavy and variable light chain (V_H_ and V_L_) against CD16 linked to the scFv of either one (BiKEs) or two (TriKEs) variable regions from other Abs that target tumour antigens. Thus, BiKEs and TriKEs are designed to enhance the interaction between tumour cells and NK cells and promote ADCC whilst minimising collateral damage to healthy cells and tissues.

BiKEs and TriKEs specific for CD16 and CD19/22 can direct NK cells for the killing of acute lymphoblastic luekaemia cells in addition to augmenting NK cell cytokine secretion [163]. Moreover, an anti-CD16xCD33 bespoke BiKE can overcome inhibitory signalling mediated by HLA class I to promote the potent cytotoxicity of primary cancer cells as well as CD33^+^ myeloid-derived suppressor cells in patients with myelodysplastic syndrome [164,165,166]. Moreover, either one of the scFvs can be replaced by a cytokine, as in TriKE constructs, to engineer a ‘TetraKE’ construct and newer generation TriKEs and TetraKEs all incorporate an IL-15 moiety that substantially enhances the function of NK cells [167,168]. BiKEs and TriKEs have distinct advantages compared to therapeutic mAbs; their smaller size results in increased biodistribution, they are non-immunogenic, and can be swiftly engineered, which alleviates many of the caveats surrounding CAR-based technologies [162].

## 6. Chemotherapy

Immunotherapies, such as checkpoint blockade, are proving to be an effective clinical approach for cancer. However, poor anti-tumour responses appear to be a major factor in the failure of cancer immunotherapy. Strategies designed to arouse anti-tumour immune responses may be of considerable benefit prior to immunotherapy and accumulating evidence suggests that immunotherapy may be more effective when combined with other treatment approaches, such as surgery, radiotherapy, and chemotherapy [169,170].

Chemotherapy agents that induce genotoxic stress or DNA replication inhibitors can upregulated the expression of NKG2DLs on target cells by activating the DDR checkpoint kinases, ATM and ATR, to promote elimination by NK cells [171]. The DDR is a program that maintains genome integrity through cell cycle arrest and activation of DNA repair, or through the induction of apoptosis or cellular senescence and permanent cell cycle arrest [172]. Most chemotherapy agents used in the clinic can trigger the DDR and treatment with the chemotherapeutic drugs; doxorubicin, etoposide, melphalan, bortezomib, and cisplatin, induced stress-induced senescence and the upregulation of ligands for DNAM-1 and NKG2DLs on multiple myeloma cells leading to NK cell activation [173].

A recent screen of several chemotherapy agents in a KRAS-mutant lung cancer mouse model identified two clinically approved cancer drugs that promoted anti-tumour immunity. Interestingly, only a combination of the two drugs, a mitogen-activated protein kinase inhibitor and a cyclin-dependent kinase 4/5 inhibitor, promoted retinoblastoma protein-mediated cellular senescence and activation of the senescence-associated secretory phenotype (SASP), which did not occur when either drug was used alone. Two SASP components, TNF-α and ICAM-I, were critically required for promoting NK cell surveillance of the drug-treated tumour cells, tumour regression and prolonged survival in the KRAS-mutant lung cancer model [174].

## 7. Conclusions

NK cell-based therapies have changed the standard of cancer care, most notably with FDA approval of rituximab for lymphoma. Current methods to unleash NK cell functions are therefore promising. However, long-term anti-tumour efficacy remains modest, particularly for solid tumours that establish an immunosuppressive microenvironment [12]. It is likely that a combination of strategies is ultimately required to improve existing NK cell therapies. Such strategies might include efforts to expand, differentiate, and maintain NK cell numbers with cytokines, such as IL-15 [175,176,177,178], and to stimulate those NK cell activation pathways most effective for the tumour type (either by checkpoint blockade and/or augmentation of activating pathways), as well as improving methods to target NK cells to tumour cells in vivo and efforts to neutralise immunosuppressive factors in the solid tumour microenvironment [12,179]. Further characterisation of the interactions within the tumour microenvironment and of NK cell receptors, particularly their ligands and checkpoints, is urgently required to improve understanding of how NK cells sense different tumour types and how this can be optimised for the clinic. Moreover, recent studies have shown that extracellular secreted or shed tumour ligands, such as PDGF-DD and MULT1, respectively, can promote NK cell activation. These data challenge the prevailing view that binding of soluble tumour-derived ligands to activating receptors invariably leads to NK cell inhibition. Thus, more basic research into the molecular basis and cell biology of activating NK cell receptor signalling in response to soluble tumour ligands, such as PDGF-DD and MULT1, is required and will inform methods to enhance NK cell targeting to tumours and stimulate their functions in vivo. For most cancers, only a subset of patients exhibit durable anti-tumour responses following immunotherapy and relapse remains a significant problem for haematological malignancies following HCST [54,119] and so strategies to exploit favourable donor immunogenetics are also warranted (e.g., KIR/HLA as well as CD16 genotypes). These latter strategies will have the added benefit of informing basic research into NK cell education and the generation of adaptive ‘memory’ NK populations. More recently, the tremendous potential of immune engagers, such as BiKEs and TriKEs, to enhance targeting through CD16 and further stimulate NK cell function with cytokines will lead to the development of a new generation of recombinant agents for NK cell-based immunotherapies. Finally, recent results have shown that chemotherapy can boost the immune response and sensitise immunologically recalcitrant tumours to immunotherapy. It will be interesting to screen combinations of clinically approved drugs for anti-tumour activity and to investigate the precise underlying molecular mechanisms for different tumour types, such as enhanced NK cell immunosurveillance.
